# Alginate Extracted from *Azotobacter chroococcum* Loaded in Selenium Nanoparticles: Insight on Characterization, Antifungal and Anticancer Activities

**DOI:** 10.3390/polym16142065

**Published:** 2024-07-19

**Authors:** Hebah A. Sindi, Ragaa A. Hamouda, Marwa S. Abdel-Hamid, Nuha M. Alhazmi

**Affiliations:** 1 Department of Biological Sciences, College of Science, University of Jeddah, Jeddah 21589, Saudi Arabia; sindi@uj.edu.sa (H.A.S.);; 2 Department of Biology, College of Sciences and Arts, Khulais, University of Jeddah, Jeddah 21959, Saudi Arabia; 3 Microbial Biotechnology Department, Genetic Engineering and Biotechnology Research Institute, University of Sadat City, Sadat City 32897, Egypt; marwa.salah@gebri.usc.edu.eg

**Keywords:** alginate, selenium nanoparticles, *Azotobacter* sp., antifungal, anticancer

## Abstract

Cancer is a threatening disease that needs strong therapy with fewer side effects. A lot of different types of chemotherapy or chemo-drugs are used in cancer treatments but have many side effects. The increasing number of antibiotic-resistant microorganisms requires more study of new antimicrobial compounds and delivery and targeting strategies. This work aims to isolate and identify *Azotobacter* sp., and extract alginate from *Azotobacter* sp. As well as fabricating selenium nanoparticles using ascorbic acid as reducing agent (As/Se-NPs), and loading extracted alginate with selenium nanoparticles (Alg-Se-NCMs). The As/Se-NPs and Alg-Se-NCMs were categorized by TEM, EDX, UV–Vis spectrophotometry, FT-IR, and zeta potential. The antifungal activities of both As/Se-NPs and Alg-Se-NCMs were investigated against some human pathogen fungi that cause skin infection such as *Aspergillus niger* (RCMB 002005), *Aspergillus fumigatus* (RCMB 002008), *Cryptococcus neoformans* (RCMB 0049001), *Candida albicans* (RCMB 005003), and *Penicillium marneffei* (RCMB 001002). The anticancer activities were determined against HCT-116 colorectal cancer and Hep G2 human liver cancer cells. UV spectra of As/Se-NPs and Alg-Se-NCMs confirmed a surface plasmon resonance at 269 and 296 nm, and zeta potential has negative charges −37.2 and −38.7 mV,. Both As/Se-NPs and Alg-Se-NCMs were hexagonal, size ranging from 16.52 to 97.06 and 17.29 to 44.2. Alg-Se-NCMs had anticancer activities against HCT-116 and HepG2. The Alg-Se-NCMs possessed the highest antifungal activities against *Cryptococcus neoformans*, followed by *Aspergillus niger*, but did not possess any activities against *Penicillium marneffei*. Alginate-capped selenium nanoparticles can be used as antifungal and anticancer treatments.

## 1. Introduction

Cancer is a dreadful worldwide disease that causes major health problems and high mortality rates among diseased persons [1]. Chemotherapy is a common treatment that kills or inhibits the growth of cancer cells [2]. Chemotherapy medications adhere to fast-growing cancer cells, also attacking healthy cells that develop quickly [3]. The limitations of the usual chemotherapy have resulted in the creation of smart nano-drugs that promise to treat cancer cells [4]. The occurrence of antibiotic-resistant isolates and bacterial biofilm formation necessitates the implementation of innovative treatment techniques [5]. 

HCT-116 colorectal cancer cells and HepG2 hepatocellular carcinoma cells are widely used in cancer research for their relevance and unique characteristics. HCT-116 cells offer insight into colorectal cancer biology, with stable genetics and varied drug sensitivity [6]. HepG2 cells, resembling hepatocytes, provide a platform for studying hepatocellular carcinoma, including drug screening and metabolism studies [7]. Both cell lines play pivotal roles in advancing our understanding of cancer and developing therapeutic strategies.

*Candida albicans*, *Aspergillus niger*, *Aspergillus fumigatus*, *Penicillium marneffei*, and *Cryptococcus neoformans* collectively contribute to a range of diseases, notably affecting individuals with compromised immune systems [8]. *Aspergillus fumigatus* and *A. niger* can cause primary and secondary cutaneous infections, presenting as nodules, ulcers, or plaques, with potential hematogenous spread leading to disseminated disease [9,10,11]. *Candida albicans*, a common opportunistic yeast, can cause cutaneous candidiasis, characterized by erythematous patches or pustules in moist areas like skin folds [12,13]. *Cryptococcus neoformans*, a fungal pathogen found in bird droppings, can cause cryptococcosis, presenting with papules, nodules, or ulcerative lesions, especially in immunocompromised patients [14]. *Penicillium marneffei*, endemic to Southeast Asia, can cause disseminated disease with skin lesions resembling molluscum contagiosum in HIV-infected individuals [15]. Timely diagnosis and appropriate treatment are crucial in managing these diverse skin infections, emphasizing the importance of targeted antifungal therapy and supportive care to improve patient outcomes and prevent complications.

Sodium alginate is a normal anionic polysaccharide acquired from several species of brown algae and has antimicrobial activities [16]. Alginate is a natural polymer exhaustively studied in the last decade for the improvement of drug delivery systems because of its non-toxicity, low cost, biocompatibility, and non-immunogenic properties [17]. 

Alginate-based nanomaterials can be used to control and target drug delivery in cancer therapy and minimize damage to healthy cells as well as against fungal infection [18]. Silver nanoparticles can be stabilized by alginate and show significant antifungal activity. Silver nanoparticles incorporated with 0.0005–0.005% sodium alginate completely inhibited *Candida albicans* viability within 24 h [19]. Usually, commercial alginates are extracted from brown algae; there is significance in the manufacture of alginate from bacteria [20]. Alginate can also be produced by some bacteria like *Azotobacter vinelandii*, *A. chroococcum*, and numerous species of Pseudomonas [21]. 

Selenium is a trace element that has antioxidant properties, often associated with health benefits in small quantities [22]. It plays a vital part in human biology, providing the proper functioning of the heart [23], cardiovascular system [24], liver [25], kidneys [26], thyroid [27], and brain [28], as well as the immune system [29] and cellular health improvement [30].

Selenium is gaining attention for its potential role in cancer treatment. When formulated as nanoparticles (Se-NPs), selenium can offer unique advantages in cancer therapy, including targeted drug delivery, reduced toxicity, and enhanced therapeutic effects [31]. It has beneficial effects on the most common types of cancers including prostate cancer [32], breast cancer [33], lung cancer [34], colon cancer [35], lymphoma [36], and leukemia [37]. 

Se-NPs also had antifungal activities against *Candida albicans* and *Aspergillus fumigates* with MICs of 70 μg/mL and 100 μg/mL, respectively [38]. Se-NPs and their combination with chitosan had antifungal activities and were suggested for use in coating fruits to eliminate postharvest pathogenic fungi [39].

Alterations within human gut microbes have been related to many cancers, including colorectal, pancreatic, head and neck, and lung, as well as benign and malignant skin illnesses [40]. More and more research are revealing the relationship and potential participation of specific fungal species, such as the commensal pathobionts Candida species, in the induction of carcinogenesis and metastasis in many organs of the human body [41]. There are association between fungal dysbiosis, through loss of beneficial fungi and increase in fungal pathogens, within colon and colorectal polyps [42].

An alginate–chitosan–lipopolysaccharide–selenium nanocomposite was able to significantly increase anti-inflammatory cytokines and decrease the release of pro-inflammatory cytokines [43]. Diverse sodium alginate–selenium nanocomposites can be used as antioxidant agents, therapeutic agents for cardiovascular issues, and in drug delivery systems [44]. A formulation based on alginate/chitosan is a suitable matrix to be used for selenium delivery in the duodenum, caecum, and colon [45].

Considering all the above, there is a vital need for continuous development in the design and progress of anti-infective and antitumor agents.

This study aims to extract alginate from Azotobacter sp., and synthesize selenium nanoparticles using ascorbic acid as a reducing agent (As/Se-NPs). Alginate derived from Azotobacter is used to stabilize selenium nanoparticles, by loading on it, to form (Alg-Se-NCMs). The effect of Alg-Se-NCMs as an anticancer treatment against HCT-116 and Hep G2 was tested. The effect of Alg-Se-NCMs against many pathogenic fungi was also tested.

## 2. Material and Methods

### 2.1. Azotobacter spp. Isolation

In a sterile test tube, one gram of soil was suspended in nine milliliters of distilled sterile water and vigorously shaken. Approximately 10 µL of each serial dilution up to 10^−3^ was distributed on a plate containing Ashby’s nitrogen-free selective media after serial dilutions were made [46]. After five days of cultivation at 28 °C, the infected plates were sub-cultured on Ashby’s media to produce pure cultures and DNA was extracted. The 16S rRNA gene of *Azotobacter* spp. Was magnified using the primers f D1 and r D1 and PCR conditions as explained by Weisburg et al. [47].

### 2.2. Azotobacter spp. Identification 

Whole genome amplification (bp) and the sequence of the 16S rRNA gene were obtained to identify *Azotobacter* spp. The obtained 16S rRNA sequences were placed in the National Center for Biotechnology Information (NCBI) GenBank nucleotide database to obtain an accession number. 

### 2.3. Phylogenetic Analysis

For confirmation of bacterial isolate identity at the species level, the obtained 16S rRNA gene sequences were subjected to a comparative analysis using the Basic Local Alignment Search Tool (BLAST) (http://www.ncbi.nlm.nih.gov/BLAST, accessed on 6 April 2024) against the GenBank database. This search identified closely related bacterial 16S rRNA gene sequences. Subsequently, the retrieved closest sequences were aligned using the CLUSTAL W software (version 1.8.3) to generate a multiple sequence alignment based on maximizing sequence identity. 

### 2.4. Alginate Extraction

Approximately 1 mL of 0.5 M EDTA-sodium salt and 0.5 mL of 5 M NaCl were mixed into a 25 mL culture broth. The mixture was added for 5 min to precipitate the cells before being centrifuged for 30 min at 38,000 rpm at 20 °C. Three-fold ice-cold isopropanol was added to the supernatant to precipitate alginates and then recovered by centrifugation at 38,000 rpm for 30 min at 4 °C. The white precipitate was washed, dried for 24 h at 60 °C, and characterized by FT-IR and ^1^H NMR [48].

### 2.5. Synthesis of Selenium Nanoparticles 

Briefly, 40 mL of sodium selenite (Na_2_SeO_3_) solution (5 mM) was placed in a flask, and then drop-wise 20 mL of ascorbic acid (vitamin C) 40 mM was added. The flask was put in a magnetic stirrer. After the color changed, the nanoparticles were collected by centrifuging at 1500 rpm for 15 min then washed three times by D.D water [49].

### 2.6. Loading Alginate with Selenium Nanoparticles (Alg-Se-NCMs)

Approximately 0.5 g of extracted alginate and 0.5 g of Se-NPs were added to 100 mL DD water, and the mixture was heated at 60–70 °C with a magnetic stirrer at 400 rpm until it turned a deep red wine color. The resulting mixture was centrifuged at 15,000 rpm for 30 min before being lyophilized.

### 2.7. Characterizations of Alginate 

Fourier transform infrared spectroscopy is a vital device that is used to identify the active groups. The extracted alginate from *Azotobacter* was analyzed using FT-IR spectroscopy and compared with standard alginic acid obtained from Sigma (sigma Egypt) Ltd, Cairo, Egypt. Dry alginate was blended with pellets of potassium bromide and the FT-IR spectra were then inspected within the range of 400–4000 cm^−1^ using a Thermo Fisher Nicolete IS10, (Waltham, MA, USA) Spectrophotometer. ^1^H NMR spectra NMR spectra (Brucker, USA, AVANCE, NEO) were acquired in 0.1% *w*/*v* solutions of alginic acid and alginate extracted from azotobacter in deuterated DMSO.

### 2.8. Characterizations of As/Se-NPs and Alg-Se-NCMs

UV–Vis spectrophotometry, Fourier-transform infrared (FT-IR) spectrometry, (Thermo Fisher Nicolet IS10, Waltham, MA, USA), energy-dispersive spectroscopy (EDS) (JEOL JSM-6510/v, Tokyo, Japan), transmission electron microscopy (TEM) (JEOL JSM-6510/v, Tokyo, Japan), and zeta potential analysis (Malvern Zeta size Nano-Zs90, Malvern, PA, USA) were carried out. The methods used in all devices are present in Supplementary Materials. 

### 2.9. Anticancer Activities

The anticancer activities of Alg-Se-NCMs on HCT-116 colorectal cancer cells, Hep G2 which is a human liver cancer cell line, and the Vero cell line (obtained from National Cancer Institute Pharmacology Unit, Cairo University) were evaluated by 3-(4,5-dimethylthiazol-2-yl)-2,5-diphenyltetrazolium bromide (MTT) assay [50].

### 2.10. Assessment of the Antifungal Activity of As/Se-NPs and Alg-Se-NCMs

The antifungal evaluation of the As/Se-NPs and Alg-Se-NCMs was performed via the inhibition method for the fungal growth of *Aspergillus fumigatus* (RCMB 002008), *Candida albicans* (RCMB 005003), *Aspergillus niger* (RCMB 002005), *Cryptococcus neoformans* (RCMB 0049001), *Penicillium marneffei* (RCMB 001002). The antifungal testing was carried out using the diffusion agar technique. A suspension of 10^6^ conidia/mL, or 1 CFU, of 5-day fungal culture of each species was primed to coverage over the solid culture medium PDA medium. Wells of 0.6 mm were created in the agar plates and embedded with 100 µL of each of As/Se-NPs and Alg-Se-NCMs, and the results were observed after 5 days of incubation. 

### 2.11. Data Analysis

The results were exposed to a one-way ANOVA for mean ± standard error of the means (n = 3) and significant differences between the means of the results were evaluated using Tukey (*p* ≤ 0.05), via SPSS software (SPSS version 16.0, SPSS Inc., Chicago, IL, USA).

## 3. Results and Discussion

### 3.1. Bacterial Identification and Phylogenetic Analysis

**Figure 1:**
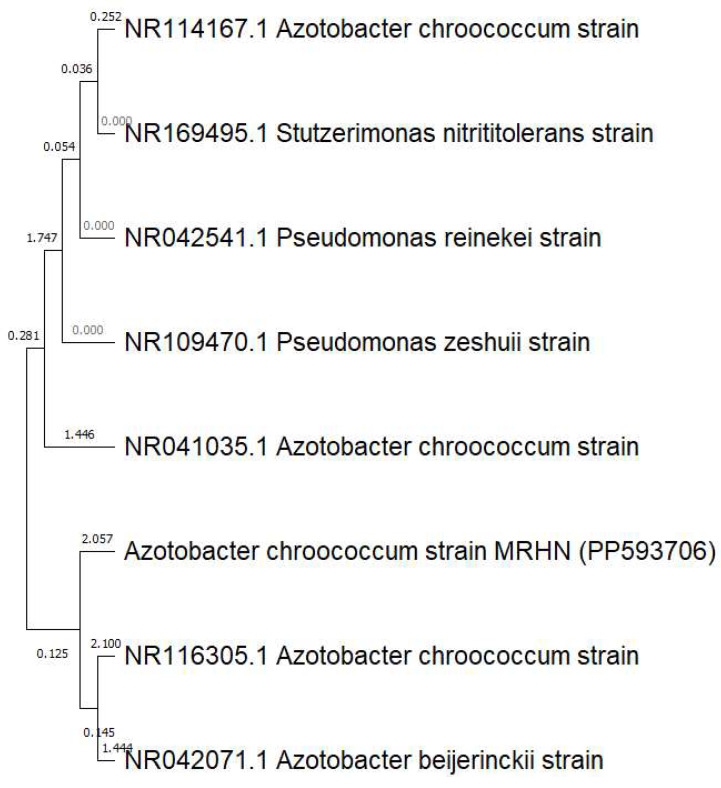
Phylogenetic analysis constructed on 16S rRNA gene comparison displaying the relationships among *A. chroococcum* MHRN (PP593706) and other closely related bacteria. The tree was assembled using the Clustal W sequence alignment tool in MEGA 11.0 Software.

*Azotobacter chroococcum* was characterized as Gram-negative, bacillus-shaped, creamy colonies with smooth convex type and circular shape on agar plates. The 16S rRNA sequence analysis showed the isolate to be *A. chroococcum*. Obtained sequences were submitted to NCBI and GenBank nucleotide sequence database and were placed under accession number MRHN (PP593706). The phylogenetic tree built using the retrieved 16S rRNA gene sequences is shown in Figure 1. This analysis effectively resolves the evolutionary relationships among the isolated *A. chroococcum* with a high degree of assurance, offering definitive insights into their taxonomic assignment.

### 3.2. Alginate Characterization

#### 3.2.1. Proton Nuclear Magnetic Resonance (^1^H NMR) Analysis 

**Figure 2:**
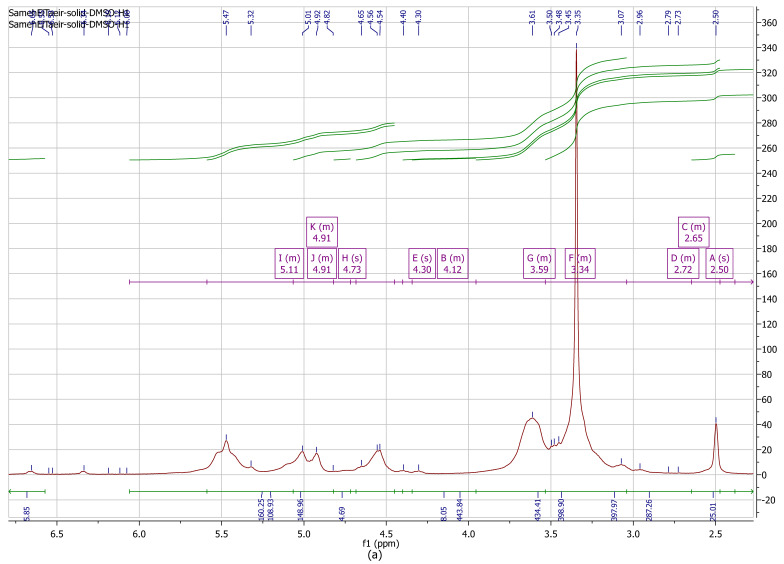

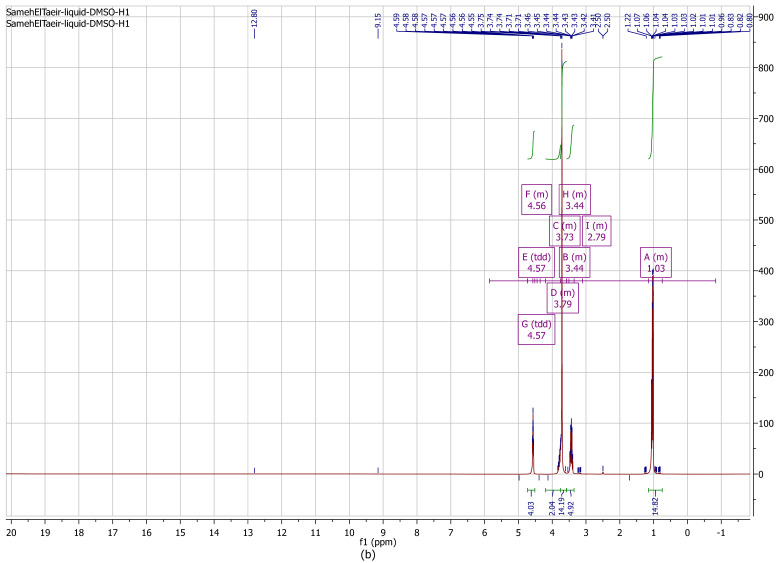
(**a**) **^1^**H NMR spectra (500 MHz) of sodium alginate (commercial); (**b**)**^1^**H NMR spectra (500 MHz) of sodium alginate solutions extracted from *A. chroococcum* MRHN (PP593706).

Hydrogen-1 NMR spectroscopy is a vital device for investigating polysaccharides’ structure, such as alginate. For the display of the structure of standard alginate, the limits for the signals were set to signal A at 2.5 ppm, signal B at 4.12, signal C at 3.59, signal D at 2.72, E at 4.3, F at 3.34, G at 3.59, H at 4.73, I at 5.11, J at 4.91, and K at 4.91 (Figure 2a). The structures of alginates determined by ^1^H NMR are shown in Figure 2. For the display of the results, the signals were set to signal A at1.03 ppm; signal B at 3.44 ppm; signal C at 3.73; signal D at 3.79 ppm; signal E at 4.57 ppm; signal F at 4.56 ppm; signal G at 4.57; signal H at 3.44 ppm, and signal I at 2.79 ppm. There were few differences between standard alginate and alginate extracted from *A. chroococcum* MHRN (PP593706). The alginate consisting of 1,4-linked β-d-mannuronic acid (M) and α-l-guluronic acid (G) can differ among algal species and also in different tissues of the same species [51]; there is a difference between algal alginate and bacterial alginate [52].

Huamani-Palomino [53] reported the chemical shifts of the mannuronic acid residue of alginate ^1^H NMR assigned at 3.74 and 3.75 ppm, with the chemical shifts for guluronic acid residue in alginate ^1^H NMR assigned at 4.46 ppm. The signal at 4.46 ppm is assigned to H-5 of guluronic units [54]. Three distinct signals of alginate are detected at 5.07, 4.70, and 4.46 ppm, and are ascribed to the anomeric proton of guluronic acid [55]. The relation of the anomeric proton in the alginate in adding to the appearance of proton signals appears between 2.79 and 4.57 ppm.

#### 3.2.2. FT-IR Analysis of Alginic Acids and Extracted from *A. chroococcum* MRHN (PP593706)

The FT-IR spectra of commercial sodium alginate and alginate extracted from *A. chroococcum* were presented in a previous study [56].

### 3.3. UV–Vis Spectroscopy Assessment

**Figure 3:**
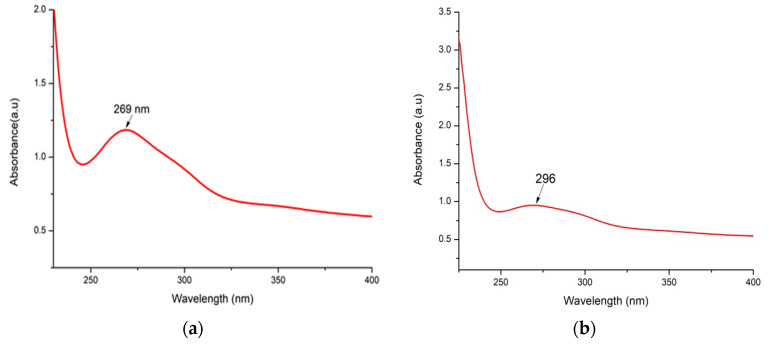
UV spectrophotometry of As/Se-NPs (**a**) and Alg-Se-NCMs (**b**).

The As/Se-NPs were identified by color modifications and investigated by a UV spectrophotometer. The assessment of the nanoparticles was promoted greatly by UV spectrophotometry. Figure 3 shows the UV spectrophotometry results for As/Se-NPs and Alg-Se-NCMs. The results clearly showed that the surface plasmon resonance was distinctly obvious at 269 and 296 nm for As/Se-NPs and Alg-Se-NCMs; the absorbance for the As/Se-NPs was observed at 1.35 a.u, and for the Alg-Se-NCMs 0.95 a.u. The change in color was due to loaded alginate on the selenium nanoparticles. Little change in color was observed in Se-NPs, when Spirulina was loaded in a Se-NP solution, suggesting that Spirulina Se-NPs dispersed in the colloid system had high stability [57].

The color of selenium nanoparticles changed from colorless to a ruby red color, having an absorption maximum ranging between 200 and 400 nm [58]. The color of sodium selenite changed when ascorbic acid (vitamin C) was added, due to the formation of selenium nanoparticles [59]. The UV–vis spectra of the biogenic selenium nanoparticles were disclosed at the surface plasmon resonance (SPR) band at 252 nm and the highest intensity was noticed at 48 h [60]. Hamouda et al. [49] reported the strong absorbance peak of selenium nanoparticles was detected at 280 nm. The decrease in the intensity of Alg-Se-NCMs may be due to the small organic molecules existing in the mixture when the added alginate was extracted from *A. chroococcum* MRHN (PP593706). The UV–vis spectroscopy revealed a peak absorption spectrum at 266 nm for selenium nanoparticles (Se-NPs) and sodium alginate-encircled selenium nanoparticles (SASe-NPs) [61].

### 3.4. FT-IR Analysis of As/Se-NPs (a) and Alg-Se-NCMs

**Figure 4:**
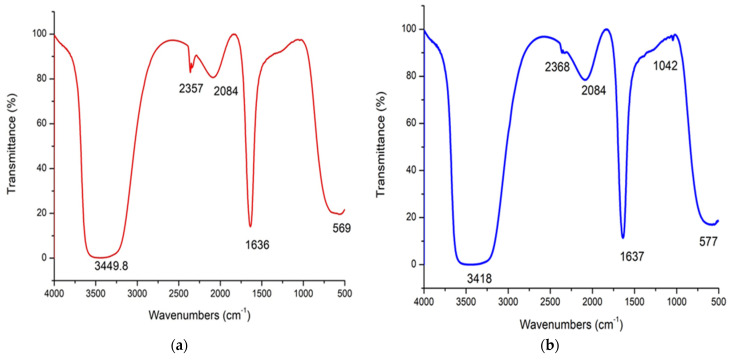
FT-IR analysis spectroscopy of As/Se-NPs (**a**) and Alg-Se-NCMs (**b**).

The FT-IR spectroscopy of the As/Se-NPs and Alg-Se-NCMs is explored in Figure 4. There are five band regions at 3449.8, 2357, 2084, 1636, and 569 cm^−1^ assigned in As/Se-NPs, while six band regions were assigned at 3418, 2368, 2084, 1637, 1042, and 577 cm^−1^ in Alg-Se-NCMs. The band at 3449.8 cm^−1^ in As/Se-NPs was shifted to 3418 cm^−1^ when alginate was loaded in Se-NPs, which represent OH stretching [44]. The band at 2357 cm^−1^ relates to the asymmetrical stretching of CO_2_ [62]. The band at 2084 cm^−1^ relates to CO molecules [63]. The band at 1636 cm^−1^ in As/Se-NPs shifted to 1637 cm^−1^ present in Alg-Se-NCMs is assigned to the amide [64]. The peak at 1024 cm^−1^ is present in Alg-Se-NCMs and appointed to C-O stretching [65]. The band at 569 shifted to 577 cm^−1^ in Alg-Se-NCMs is attributed to OH out-of-plane bending of the molecule [66].

### 3.5. Zeta Potential 

**Figure 5:**
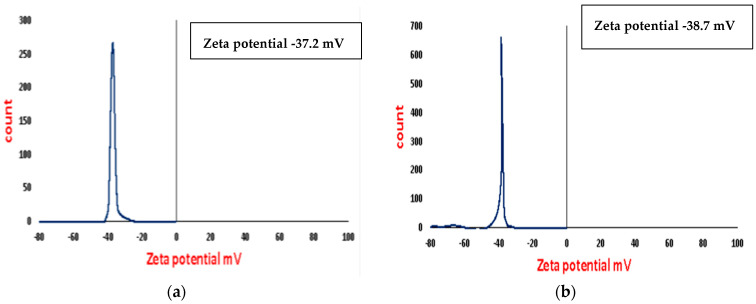
Zeta potential analysis of As/Se-NPs (**a**) and Alg-Se-NCMs (**b**).

From the zeta potential results found in Figure 5a,b, it is clear that the surface charge of the As/Se-NPs is −37.2 mV and in the case of Alg-Se-NCMs is −38.7. These results show that both As/Se-NPs and Alg-Se-NCMs are highly stable due to the electrostatic repulsion [67]. The negatively charged metal nanoparticles possessed electrostatic repulsive forces and lowered the aggregation [49]. The surface charge of biogenic Se-NPs has a negative charge of −25.7 mV [68]. The zeta potential analysis displayed a sharp peak at −71 mV, representing the high stability of the Selenium nanoparticles dispersed in the medium [69]. The results validate that the negative charges were increased when alginate capped selenium nanoparticles. The stability of the Se-NPs was increased when capped by different polymers like alginate which can form many chemical bonds with the selenium nanoparticles [70].

### 3.6. X-ray Diffraction (XRD)

**Figure 6:**
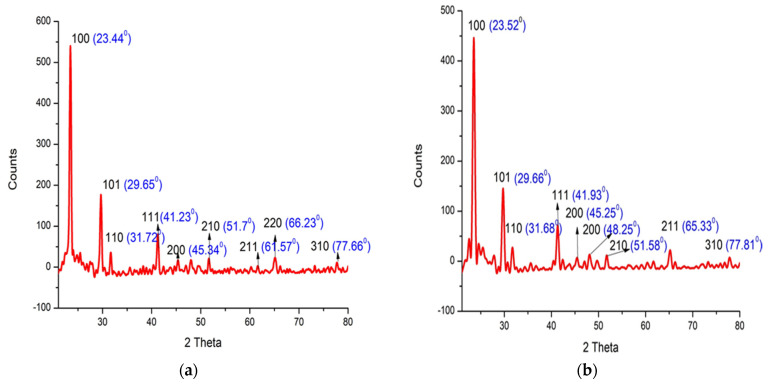
X-ray diffraction of As/Se-NPs (**a**) and Alg-Se-NCMs (**b**).

X-ray diffraction was used to explore the size and crystallization of As/Se-NPs and Alg-Se-NCMs. The results showed that the peak positions with 2Ө values of As/Se-NPs 23.44, 29.65, 31.72, 41.23, 45.34, 51.7, 61.57, 66.23, and 77.66 correspond to lattice planes (hkl) 100, 101, 110, 111, 200, 210, 211, 220, and 310 (Figure 6a). A small modification in the 2Ө position was obtained when selenium nanoparticles were capped by alginate (Alg-Se-NCMs) (Figure 6b). The peak positions of 2Ө values of Alg-Se-NCMs were recorded at 23.52, 29.66, 31.68, 41.93, 45.25, 48.25, 51.58, 65.33, 77.81 corresponding to lattice planes (hkl) 100, 101, 110, 111, 200, 200, 210, 211, and 310. The XRD proved the nanoparticles are selenium, crystalline, and hexagonal in shape. The results of XRD detected that the selenium nanoparticles were crystalline and rhombohedral in shape [49]. The diffraction peaks of selenium disagree with the 2θ angles listed for a hexagonal phase [71]. Chemically synthesized selenium nanoparticles are regular hexagonal prisms [72]. Green-synthesized selenium nanoparticles are well-crystallized elemental nanoparticles with a hexagonal structure [73].

### 3.7. Differential Scanning Calorimetry (DSC) and Thermogravimetric Analysis (TGA) of Alg-Se-NCMs

**Figure 7:**
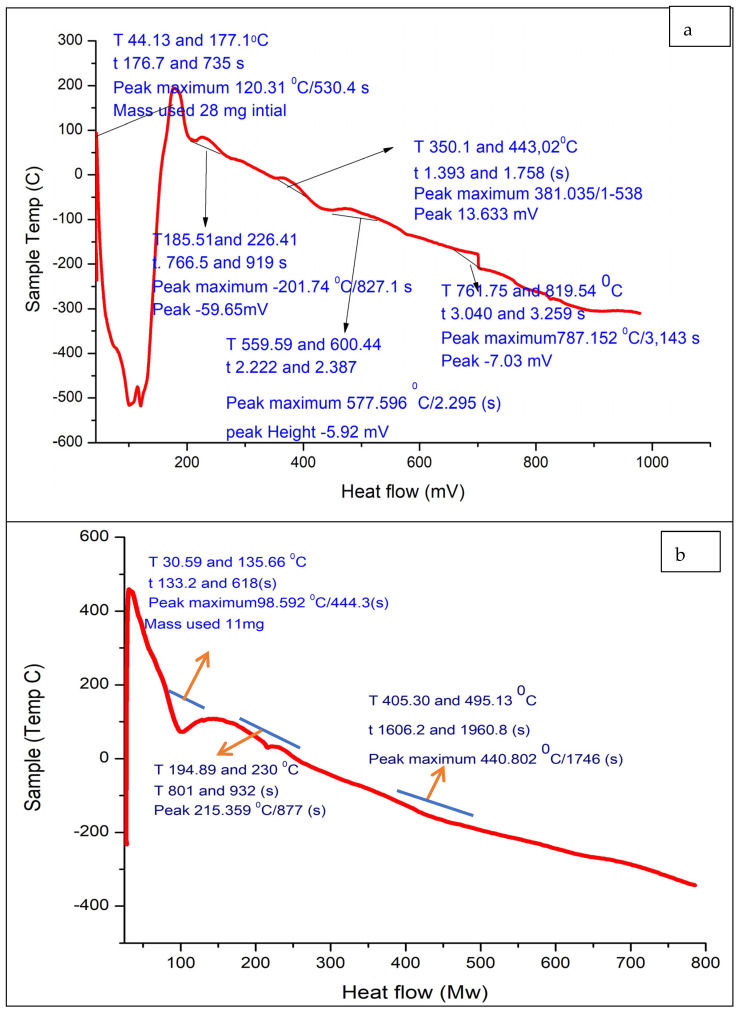
DSC analysis of Alg-Se-NCMs (**a**) and As-Se-NPS (**b**).

The melting temperature (Tm) and glass transition temperature (T*g*) of the of As/Se-NPs and Alg-Se-NCMs were evaluated to determine their thermal behavior. The heat absorbed or emitted by the Alg-Se-NCMs as a function of time or temperature is measured by DSC. The DSC curves derived from Alg-Se-NCMs and As/Se-NPs are illustrated in Figure 7a,b. The Alg-Se-NCMs’ DSC curve shows five peaks in the curve (Figure 7a). The first endothermic peak, ranging from 44.13 to 177.1 °C, represents the thermal effect of dehydration and water loss from the Alg-Se-NCMs sample; then four exothermic peaks appear with temperatures ranging from 185.51 to 226.41, 350.1 to 443.02, 559.59 to 600.44, and 761.75 to 819.54 °C, respectively. The water loss was limited to the first endothermic peak below 100 °C [74]. The exothermic peak at 169.5 °C was attributed to water loss due to the hydrophilic properties of the alginate polymer [75]. The glass transition temperature of water evaporation caused the peak temperature to fluctuate between 75 and 120 °C [76]. The four exothermic peaks which appeared may be accredited to the decomposition of alginate and selenium nanoparticles. The exothermic peaks close to 240 and 300 °C are attributed to the decomposition of the polymer [77]. The exothermic peak, which ranged from 225 and 317.12 °C, was ascribed to the thermal decomposition of the polymer’s nanocomposites [78]. The two peaks close to 559.59 and 761.75 may be attributed to the formation of carbonaceous material after the decomposition of alginate. The decay of polymers around 400 °C was described as a carbonaceous material [77]. The peak was temperatures above 525 °C, assigned to the formation of metal carbonates [79]. The results in Figure 7b demonstrate the DSC curve of As/Se-NPs which shows three endothermic peaks; the first endothermic peak ranging from 30.59 to 135.66 represents the thermal effect of water loss from the As/Se-NP sample, followed by two endothermic peaks with temperatures ranging from 194.89 to 230 °C, and from 405.3 to 495.13 °C.

The selenium nanoparticles lose their crystalline nature in the first thermal run itself, and the transition could be ascribed to enhancements in the crystallinity of the nanoparticles. The DSC thermogram of the selenium sample shows a melting peak at 217 °C, without any such exothermic peak [80]. The DSC thermogram of the standard selenium sample did not display any exothermic peak [81].

**Figure 8:**
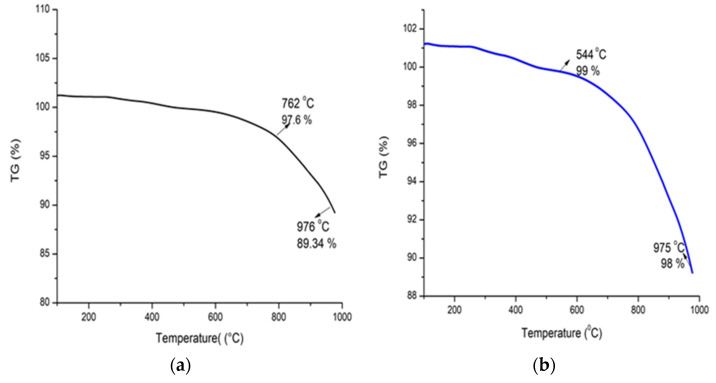
TGA analysis of Alg-Se-NCMs (**a**) and As-Se-NPS (**b**).

The thermo-gravimetric degradation curve of the Alg-Se-NCMs in the percentage of the mass in the initial sample altering on the temperature is presented in Figure 8a. The initial weight of the sample was 1 mg and slightly decreased when the temperature increased to 762 °C, with a loss of 2.4%. A second weight loss happened when the temperature increased to 976 °C, and 10.66% of the initial weight was lost. The thermo-gravimetric degradation curve of the As-Se-NPS is shown in Figure 8b. The initial degradation was made at 544 °C, and the percentage of weight loss was 1%; the second weight loss of 2% occurred at 975 °C. Decarboxylation and dehydroxylation occur in the degradation route of iron alginate at temperatures ranging from 158 to 400 °C which is close to H_2_O and CO_2_ evolutions [81]. Heating carboxymethyl starch-capped selenium nanoparticles to 700 °C resulted in carbonization and ash construction [82].

**Figure 9:**
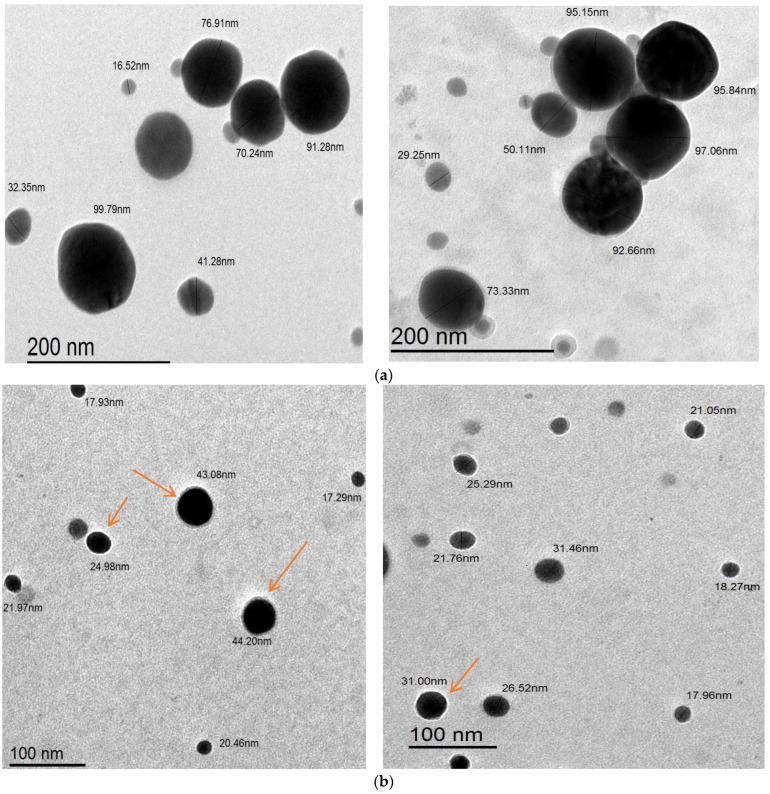
Transmission electron microscopy (TEM) images of As/Se-NPs (**a**) and Alg-Se-NCMs (**b**); the arrows indicate alginate-capped selenium nanoparticles.

The results in Figure 9a,b show the images taken by transmission electron microscopy (TEM) of both As/Se-NPs and Alg-Se-NCMs. The images demonstrate that the morphological structure of both As/Se-NPs and Alg-Se-NCMs was spherical in shape. The size of As/Se-NPs ranged from 16.52 to 97.06 while the size of Alg-Se-NCMs was less and ranged from 17.29 to 44.2. These results are in agreement with others which demonstrated that the size of alginate-capped Se-NPs was 38.94 nm [83]. The results demonstrate both As/Se-NPs and Alg-Se-NCMs are poly-dispersed, hexagonal in shape, and well distributed. The arrows in Figure 8b point to the shell core of alginate around the selenium nanoparticles. The capping of metal nanoparticles by polymers is the critical method for ensuring the nanoparticles’ stability [84]. 

### 3.8. Energy-Dispersive X-ray Measurements

**Figure 10:**
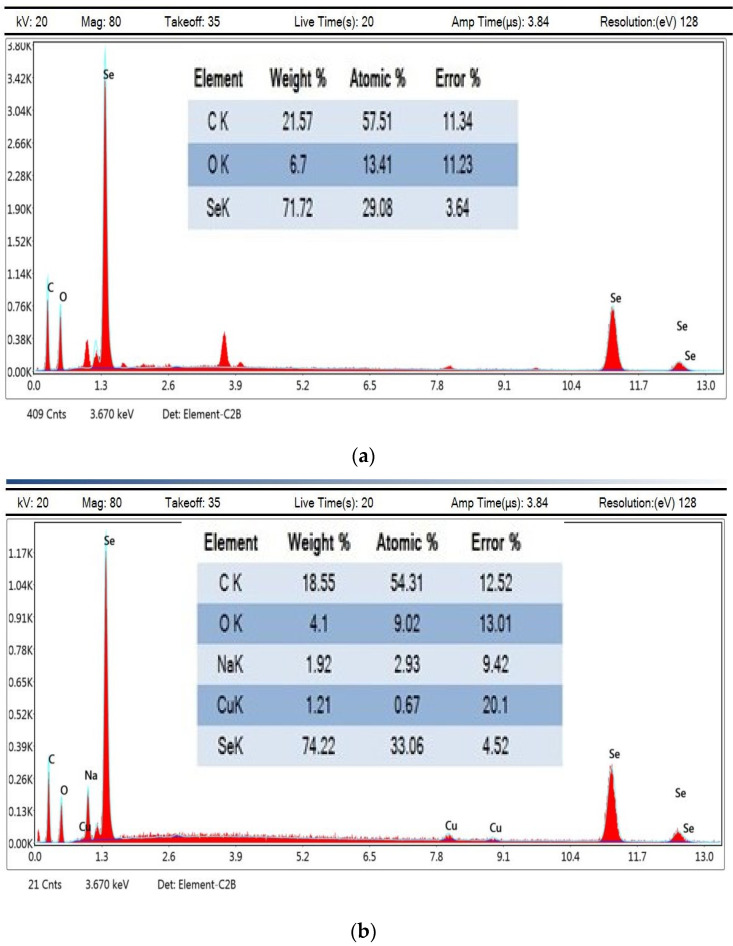
EDX analysis of As/Se-NPs (**a**) and Alg-Se-NCMs (**b**).

Energy-dispersive X-ray spectroscopy is used to investigate the relative profusion of various elements in a given sample. The EDX analysis of As/Se-NPs confirmed the occurrence of selenium, carbon, and oxygen, by weight % 71.72, 21.57, and 6.7 (Figure 10a). Figure 10b demonstrates the presence of selenium, carbon, oxygen, sodium, and copper with percentage weights 74.22, 18.55, 4.1, and 1.21, respectively. The presence of sodium may relate to the sodium alginate that capped the selenium nanoparticles. The EDX analysis showed that the nanostructures were founded solely on selenium, with the major weight being extant at 1.4 keV [49]. The results obtained by EDX demonstrate the purity of the selenium nanoparticles [85]. 

### 3.9. Antifungal Activities

**Figure 11:**
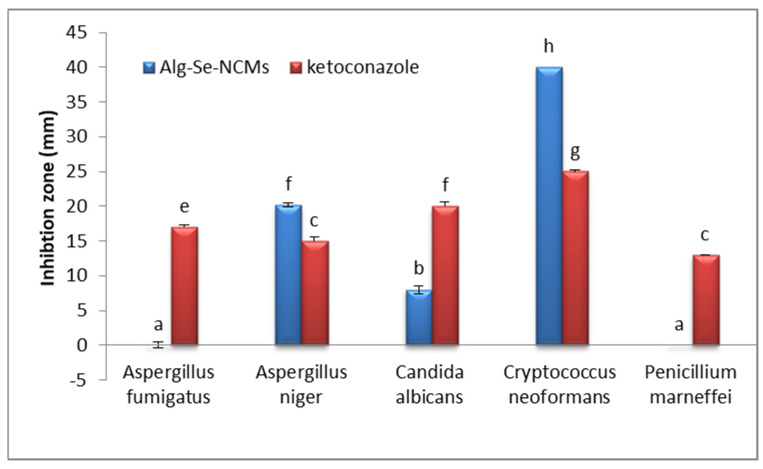
Inhibition zones (mm) of Alg-Se-NCMs and ketoconazole against fungal pathogenic strains. (Different letters denote significant values).

The results in Figure 11 illustrate the antifungal activities of both As/Se-NPs and Alg-Se-NCMs compared with ketoconazole (an antifungal medicine). The results denoted that both As/Se-NPs and Alg-Se-NCMs possessed antifungal activities against *Aspergillus niger* (RCMB 002005), *Candida albicans* (RCMB 005003), and *Cryptococcus neoformans* (RCMB 0049001), while no effects were apparent against *Penicillium marneffei* (RCMB 001002). The major effects of As/Se-NPs and Alg-Se-NCMs were obtained against *Cryptococcus neoformans* (RCMB 0049001) with inhibition zones of 40 and 39 mm, respectively, compared to ketoconazole. The As/Se-NPs possessed antifungal activities against *Aspergillus fumigatus* (RCMB 002008), while no effect appeared when using Alg-Se-NCMs against the same fungus. Biogenic selenium nanoparticles have antifungal activity against *A. fumigatus* and *C. albicans* [38]. Biogenic Se-NPs possessed antisporulant and antifungal activities against the black fungus *Aspergillus niger* [86]. Selenium nanoparticles synthesized by ascorbic acid indicated antifungal activity against all examined fungi, *Cephalosporium acremonium*, *Botrytis cinerea*, and *Fusarium semitectum* [49]. Selenium nanoparticles coated with alginate well inhibited both *Neopestalotiopsis rosae* and *Fusarium oxysporum* [87]. There are many suggested mechanisms involved in the effects of nanoparticles against fungi. The suggested mechanisms are the following: (1) changes in the fungal cell wall, involving surface shrinkage, cell wall aggregation, pore/pit formation, and general distortion; (2) cell wall destruction caused by DNA leakage out of the cell, or nanomaterial inside the cell can insert with nucleic acids intra-cellularly; (3) nanoparticles in combination with biomolecules,, such as proteins, negatively-charged lipids, and nucleic acids, wither the cell; (4) nanoparticles can inhibit mycelial growth, and harm conidia and spores, which can bind because they have more surface points and fit better into conidial surfaces; (5) nanoparticles can induce ROS generation and activity, as ROS liberation can play a role in antifungal effects; (6) protein levels and gene expression can be altered by nanoparticles [88].

### 3.10. Anticancer Activities

**Figure 12:**
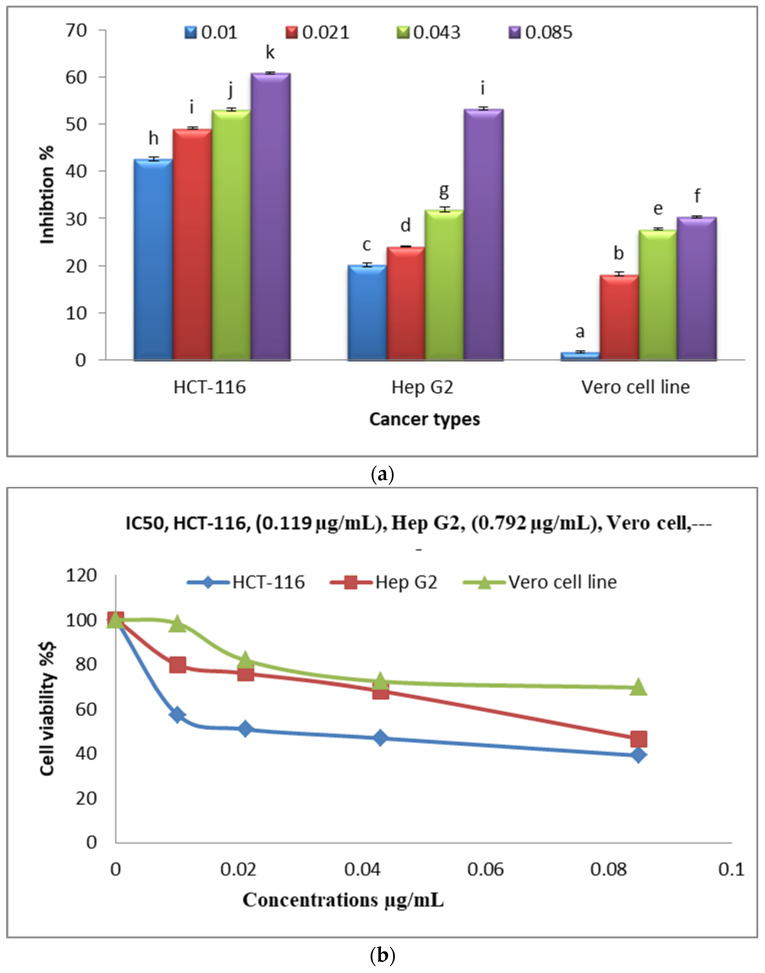
(**a**) Anticancer activities of various concentrations of Alg-Se-NCMs (µg·mL^−1^) against HCT-116 colorectal cancer cells, Hep G2 human liver cancer cell line, and Vero cell line; (**b**) HCT-116 colorectal cancer cells, Hep G2 human liver cancer cell line, and Vero cell line that were pre-treated with Alg-Se-NCMs (different letters mean significant values).

Figure 12a shows the anticancer activities of various concentrations of Alg-Se-NCMs on the HCT-116 colorectal cancer cells, Hep G2 (human liver cancer) cells, and the Vero cell line. Alg-Se-NCMs have good results against HCT-116 colorectal cancer cells, followed by Hep G2 (human liver cancer) cells, which had inhibition percentages of 60.9 and 53.3 at Alg-Se-NCMs concentrations of 0.085 µg/mL. A low level of negative effect was obtained with the Vero cell line. The results in Figure 12b display the cell viability and IC50 of HCT-116 colorectal cancer cells, Hep G2 (human liver cancer) cells, and the Vero cell line after being treated with different concentrations of Alg-Se-NCMs. The results show the IC50 of HCT-116 is 0.119 µg/mL, Hep G2 is 0.792 µg/mL, and no toxicity against Vero cells. The chitosan/selenium @ olive oil nanocomposites possessed anticancer activities against Caco2, MCF7, and HepG2 which had IC50 values of 117.99, 99.1, and 123.05 µg/mL, respectively [89]. Selenium nanoparticles were evaluated as anticancer agents versus six tumor cells [90]. Berberine-loaded selenium nanoparticles revealed excellent anticancer efficacy against HepG2 in comparison to unprocessed Berberine extract [91]. Chitosan-stabilized selenium nanoparticles inhibited the expression of cyclin-independent kinase 1 [92].

Lentinan Se-NPs stimulated apoptosis of HCT-116 cells through motivating a mitochondria-intermediated apoptotic pathway. Meanwhile, lentinan Se-NPs prompted cell cycle arrest at G0/G1 phase in HCT-116 cells through variation of cell cycle regulatory proteins which indicated that lentinan Se-NPs possessed the highest possibility of application in the treatment of colorectal cancer [35]. Selenium nanoparticles encapsulated by chitosan (Ch/Se/NPs) enhanced anticancer activities against cancer cells of human colorectal carcinoma (HCT116), human liver carcinoma (HepG-2), and human breast adenocarcinoma MCF7 [93].

## 4. Conclusions

*Azotobacter chroococcum* (PP593706) can be used for the production of alginate. Hydrogen-1 NMR spectroscopy validated the alginate formed by *A. chroococcum*. The synthesis of As/Se-NPs and Alg-Se-NCMs was proved by UV spectroscopy. FT-IR analysis proved there are small modifications in the peak positions of As/Se-NPs and Alg-Se-NCMs. The surface charges are negative and demonstrate the As/Se-NPs and Alg-Se-NCMs are stable. TEM images demonstrate the hexagonal shape. Thermography analysis demonstrates Alg-Se-NCMs are thermally stable. Alg-Se-NCMs possessed antifungal activities against *Cryptococcus neoformans*, *Candida albicans*, and *Aspergillus niger*. Alg-Se-NCMs possessed anticancer activities against HCT 116 and HepG-2 under in vitro conditions, but had no effects against the Vero cell line.

## Data Availability

The datasets spent and/or analyzed during this study are available from the corresponding author upon reasonable request.

## Supplementary Materials

The following supporting information can be downloaded at: https://www.mdpi.com/article/10.3390/polym16142065/s1.

## References

[1] American Cancer Society (2017). Cancer Facts & Figures 2017. https://www.cancer.org/research/cancer-facts-statistics/all-cancer-facts-figures/cancer-facts-figures-2017.html.

[2] Zhang W., Zhang Z., Zhang Y. (2011). The Application of Carbon Nanotubes in Target Drug Delivery Systems for Cancer Therapies. Nanoscale Res. Lett..

[3] Ahmad S.S., Reinius M.A., Hatcher H.M., Ajithkumar T.V. (2016). Anticancer Chemotherapy in Teenagers and Young Adults: Managing Long Term Side Effects. BMJ.

[4] Hossen S., Hossain M.K., Basher M.K., Mia M.N.H., Rahman M.T., Uddin M.J. (2019). Smart Nanocarrier-Based Drug Delivery Systems for Cancer Therapy and Toxicity Studies: A Review. J. Adv. Res..

[5] Asadpoor M., Ithakisiou G.-N., van Putten J.P.M., Pieters R.J., Folkerts G., Braber S. (2021). Antimicrobial Activities of Alginate and Chitosan Oligosaccharides against *Staphylococcus aureus* and Group B *Streptococcus*. Front. Microbiol..

[6] Zhou J.-Y., Chen M., Ma L., Wang X., Chen Y.-G., Liu S.-L. (2016). Role of CD44high/CD133high HCT-116 Cells in the Tumorigenesis of Colon Cancer. Oncotarget.

[7] Arzumanian V.A., Kiseleva O.I., Poverennaya E.V. (2021). The Curious Case of the HepG2 Cell Line: 40 Years of Expertise. Int. J. Mol. Sci..

[8] Deepa A., Nair B., Sivakumar T., Joseph A. (2014). Uncommon Opportunistic Fungal Infections of Oral. J. Oral Maxillofac. Pathol..

[9] Mohapatra S., Xess I., Swetha J., Tanveer N., Asati D., Ramam M., Singh M. (2009). Primary Cutaneous Aspergillosis due to *Aspergillus niger* in an Immunocompetent Patient. Indian J. Med. Microbiol..

[10] Kanaujia R., Singh S., Rudramurthy S.M. (2023). Aspergillosis: An Update on Clinical Spectrum, Diagnostic Schemes, and Management. Curr. Fungal Infect. Rep..

[11] Merad Y., Derrar H., Belmokhtar Z., Belkacemi M. (2021). *Aspergillus* Genus and Its Various Human Superficial and Cutaneous Features. Pathogens.

[12] López-García B., Lee P.H.A., Yamasaki K., Gallo R.L. (2005). Anti-Fungal Activity of Cathelicidins and Their Potential Role in *Candida albicans* Skin Infection. J. Investig. Dermatol..

[13] Halverstam C., Cohen S.R. (2018). Cutaneous Candidiasis and Chronic Mucocutaneous Candidiasis. Treatment of Skin Disease.

[14] Morales E.G., Guidi M., Peterka T., Rabufetti A., Blum R., Mainetti C. (2021). Primary Cutaneous Cryptococcosis due to *Cryptococcus neoformans* in an Immunocompetent Host Treated with Itraconazole and Drainage: Case Report and Review of the Literature. Case Rep. Dermatol..

[15] Wong S.Y.N., Wong K.F. (2011). *Penicillium marneffei* Infection in AIDS. Pathol. Res. Int..

[16] Shalumon K.T., Anulekha K.H., Nair S.V., Nair S.V., Chennazhi K.P., Jayakumar R. (2011). Sodium Alginate/Poly(Vinyl Alcohol)/Nano ZnO Composite Nanofibers for Antibacterial Wound Dressings. Int. J. Biol. Macromol..

[17] Spadari C.d.C., Lopes L.B., Ishida K. (2017). Potential Use of Alginate-Based Carriers as Antifungal Delivery System. Front. Microbiol..

[18] Iravani S., Varma R.S. (2022). Alginate-Based Micro- and Nanosystems for Targeted Cancer Therapy. Mar. Drugs.

[19] Kubyshkin A., Chegodar D. (2016). Antimicrobial Effects of Silver Nanoparticles Stabilized in Solution by Sodium Alginate. Biochem. Mol. Biol. J..

[20] Clementi F. (1997). Alginate Production by *Azotobacter vinelandii*. Crit. Rev. Biotechnol..

[21] Sabra W., Zeng A.P., Deckwer W.D. (2001). Bacterial Alginate: Physiology, Product Quality and Process Aspects. Appl. Microbiol. Biotechnol..

[22] Zhang F., Li X., Wei Y. (2023). Selenium and Selenoproteins in Health. Biomolecules.

[23] Shengyu C., Yinhua L., Yuanhong L., Jinbo Z., Can F., Hao X., Changjiang Z. (2022). Selenium Alleviates Heart Remodeling through Sirt1/AKT/GSK-3β Pathway. Int. Immunopharmacol..

[24] Shimada B.K., Alfulaij N., Seale L.A. (2021). The Impact of Selenium Deficiency on Cardiovascular Function. Int. J. Mol. Sci..

[25] Wang Y., Liu B., Wu P., Chu Y., Gui S., Zheng Y., Chen X. (2022). Dietary Selenium Alleviated Mouse Liver Oxidative Stress and NAFLD Induced by Obesity by Regulating the KEAP1/NRF2 Pathway. Antioxidants.

[26] Zhang X., Wang Q., Zhang J., Song M., Shao B., Han Y., Yang X., Li Y. (2021). The Protective Effect of Selenium on T-2-Induced Nephrotoxicity Is Related to the Inhibition of ROS-Mediated Apoptosis in Mice Kidney. Biol. Trace Elem. Res..

[27] Ventura M., Melo M., Carrilho F. (2017). Selenium and Thyroid Disease: From Pathophysiology to Treatment. Int. J. Endocrinol..

[28] Torres D.J., Alfulaij N., Berry M.J. (2021). Stress and the Brain: An Emerging Role for Selenium. Front. Neurosci..

[29] Arthur J.R., McKenzie R.C., Beckett G.J. (2003). Selenium in the Immune System. J. Nutr..

[30] Shalihat A., Hasanah A.N., Mutakin, Lesmana R., Budiman A., Gozali D. (2021). The Role of Selenium in Cell Survival and Its Correlation with Protective Effects against Cardiovascular Disease: A Literature Review. Biomed. Pharmacother..

[31] Li T., Xu H. (2020). Selenium-Containing Nanomaterials for Cancer Treatment. Cell Rep. Phys. Sci..

[32] Liao G., Tang J., Wang D., Zuo H., Zhang Q., Liu Y., Xiong H. (2020). Selenium Nanoparticles (SeNPs) Have Potent Antitumor Activity against Prostate Cancer Cells through the Upregulation of MiR-16. World J. Surg. Oncol..

[33] Khaledizade E., Tafvizi F., Jafari P. (2024). Anti-Breast Cancer Activity of Biosynthesized Selenium Nanoparticles Using *Bacillus coagulans* Supernatant. J. Trace Elem. Med. Biol..

[34] Liu S., Wei W., Wang J., Chen T. (2023). Theranostic Applications of Selenium Nanomedicines against Lung Cancer. J. Nanobiotechnol..

[35] Gao X., Yao Y., Chen X., Lin X., Yang X., Ho C.-T., Li B., Chen Z. (2022). Lentinan-Functionalized Selenium Nanoparticles Induce Apoptosis and Cell Cycle Arrest in Human Colon Carcinoma HCT-116 Cells. Front. Nutr..

[36] Gautam P.K., Kumar S., Tomar M.S., Singh R.K., Acharya A., Kumar S., Ram B. (2017). Selenium Nanoparticles Induce Suppressed Function of Tumor Associated Macrophages and Inhibit Dalton’s Lymphoma Proliferation. Biochem. Biophys. Rep..

[37] Jin Y., Cai L., Yang Q., Luo Z., Liang L., Liang Y., Wu B., Ding L., Zhang D., Xu X. (2020). Anti-Leukemia Activities of Selenium Nanoparticles Embedded in Nanotube Consisted of Triple-Helix β-d-Glucan. Carbohydr. Polym..

[38] Shakibaie M., Salari Mohazab N., Ayatollahi Mousavi S.A. (2015). Antifungal Activity of Selenium Nanoparticles Synthesized by Bacillus Species Msh-1 against *Aspergillus fumigatus* and *Candida albicans*. Jundishapur J. Microbiol..

[39] Salem M.L., Abd-Elraoof W.A., Tayel A.A., AlZuaibr F.M., Abonama O.M. (2022). Antifungal Application of Biosynthesized Selenium Nanoparticles with Pomegranate Peels and Nanochitosan as Edible Coatings for Citrus Green Mold Protection. J. Nanobioil..

[40] Squarzanti D.F., Zavattaro E., Pizzimenti S., Amoruso A., Savoia P., Azzimonti B. (2020). Non-Melanoma Skin Cancer: News from microbiota research. Crit. Rev. Microbiol..

[41] Huët M.A.L., Lee C.Z., Rahman S. (2022). A review on association of fungi with the development and progression of carcinogenesis in the human body. Curr. Res. Microb. Sci..

[42] Gao R., Kong C., Li H., Huang L., Qu X., Qin N., Qin H. (2017). Dysbiosis signature of mycobiota in colon polyp and colorectal cancer. Eur. J. Clin. Microbiol. Infect. Dis..

[43] Bagheri-Josheghani S., Bakhshi B. (2022). Formulation of selenium nanoparticles encapsulated by alginate-chitosan for controlled delivery of *Vibrio cholerae* LPS: A novel delivery system candidate for nanovaccine. Int. J. Biol. Macromol..

[44] Naveenkumar S., Alagumanikumaran N., Kaviyarasu K., Muthukumaran A. (2024). Influence of Encapsulated Sodium Alginates and Pectin on Selenium Nanoparticles and Efficient Cardioprotective Effect in C2C12 Cell Line. J. Nanoparticle Res..

[45] Cavalu S., Prokisch J., Laslo V., Vicas S. (2017). Preparation, structural characterisation and release study of novel hybrid microspheres entrapping nanoselenium, produced by green synthesis. IET Nanobiotechnol..

[46] Stella M., Suhaimi M. (2010). Selection of Suitable Growth Medium for Free-Living Diazotrophs Isolated from Compost. J. Trop. Agric. Food Sci..

[47] Weisburg W.G., Barns S.M., Pelletier D.A., Lane D.J. (1991). 16S Ribosomal DNA Amplification for Phylogenetic Study. J. Bacteriol..

[48] Abdel-Hamid M.S., Saad M.W., Badawy G.A., Hamza H.A., Haroun A.A. (2018). Synthesis and Examination of Hydroxyapatite Nanocomposites Based on Alginate Extracted from *Azotobacter chroococcum* New Strain MWGH ShKB In Vitro. Biosci. Res..

[49] Hamouda R.A., Abdel-Hamid M.S., Hagagy N., Nofal A.M. (2024). The Potent Effect of Selenium Nanoparticles Insight into: The Antifungal Activity and Preservation of Postharvest Strawberries from Gray Mold Diseases. J. Sci. Food Agric..

[50] Fertah M., Belfkira A., Taourirte M., Brouillette F. (2017). Extraction and characterization of sodium alginate from Moroccan *Laminaria digitata* brown seaweed. Arab. J. Chem..

[51] Aarstad O.A., Stanisci A., Sætrom G.I., Tøndervik A., Sletta H., Aachmann F.L., Skjåk-Bræk G. (2019). Biosynthesis and function of long guluronic acid-blocks in alginate produced by *Azotobacter vinelandii*. Biomacromolecules.

[52] Mosmann T. (1983). Rapid Colorimetric Assay for Cellular Growth and Survival: Application to Proliferation and Cytotoxicity Assays. J. Immunol. Methods.

[53] Huamani-Palomino R.G., Córdova B.M., Pichilingue L. E.R., Venâncio T., Valderrama A.C. (2021). Functionalization of an Alginate-Based Material by Oxidation and Reductive Amination. Polymers.

[54] Salomonsen T., Jensen H.M., Stenbæk D., Engelsen S.B. (2008). Rapid Determination of Alginate Monomer Compostion Using Raman Spectroscopy and Chemometrics. R. Soc. Chem..

[55] Salomonsen T., Jensen H.M., Larsen F.H., Steuernagel S., Engelsen S.B. (2009). Alginate Monomer Composition Studied by Solution- and Solid-State NMR—A Comparative Chemometric Study. Food Hydrocoll..

[56] Sindi H.A., Hamouda R.A., Alhazmi N.M., Abdel-Hamid M.S. (2024). Functionalized gold nanoparticles coated with bacterial alginate and their antibacterial and anticancer activities. Green Process. Synth..

[57] Ramamurthy C.H., Sampath K.S., Arunkumar P., Kumar M.S., Sujatha V., Premkumar K., Thirunavukkarasu C. (2013). Green Synthesis and Characterization of Selenium Nanoparticles and its Augmented Cytotoxicity with Doxorubicin on Cancer Cells. Bioprocess Biosyst. Eng..

[58] Sani-e-Zahra, Iqbal M.S., Abbas K., Qadir M.I. (2022). Synthesis, Characterization and Evaluation of Biological Properties of Selenium Nanoparticles from *Solanum lycopersicum*. Arab. J. Chem..

[59] Anu K., Devanesan S., Prasanth R., AlSalhi M.S., Ajithkumar S., Singaravelu G. (2020). Biogenesis of Selenium Nanoparticles and Their Anti-Leukemia Activity. J. King Saud Univ. Sci..

[60] Ghazali N.M., Mazuki N.F., Samsudin A.S. (2021). Characterization of Biopolymer Blend-Based on Alginate and Poly (Vinyl Alcohol) as an Application for Polymer Host in Polymer Electrolyte. IOP Conf. Ser. Mater. Sci. Eng..

[61] Huang N., Wang J. (2009). A TGA-FTIR Study on the Effect of CaCO_3_ on the Thermal Degradation of EBA Copolymer. J. Anal. Appl. Pyrolysis.

[62] Ivanova E., Mihaylov M., Thibaultstarzyk F., Daturi M., Hadjiivanov K. (2005). New Type of Rhodium Gem-Dicarbonyls Formed in Rh-ZSM-5: An FTIR Spectroscopy Study. J. Catal..

[63] Tareb R., Bernardeau M., Amiel C., Vernoux J.-P. (2017). Usefulness of FTIR Spectroscopy to Distinguish Rough and Smooth Variants of *Lactobacillus farciminis* CNCM-I-3699. FEMS Microbiol. Lett..

[64] Hamouda R.A., Almaghrabi F.Q., Alharbi O.M., Al-Harbi A.D.M., Alsulami R.M., Alhumairi A.M. (2024). Antifungal Activities of Biogenic Silver Nanoparticles Mediated by Marine Algae: In Vitro and In Vivo Insights of Coating Tomato Fruit to Protect against *Penicillium italicum* Blue Mold. Mar. Drugs.

[65] Arjunan V., Anitha R., Thenmozhi S., Marchewka M.K., Mohan S. (2016). Potential Energy Profile, Structural, Vibrational and Reactivity Descriptors of Trans-2-Methoxycinnamic Acid by FTIR, FT-Raman and Quantum Chemical Studies. J. Mol. Struct..

[66] Alghanmi R.M., Hamouda R.A., Al-Moubaraki A.H., Allouzi A.A., Abuelmagd M.A. (2024). Biofabrication of Silver Nanoparticles Using *Uncaria tomentosa* L.: Insight into Characterization, Antibacterial Activities Combined with Antibiotics, and Effect on *Triticum aestivum* Germination. Green Process. Synth..

[67] Suriyakalaa U., Antony J.J., Suganya S., Siva D., Sukirtha R., Kamalakkannan S., Pichiah P.B.T., Achiraman S. (2013). Hepatocurative Activity of Biosynthesized Silver Nanoparticles Fabricated Using Andrographis Paniculata. Colloids Surf. B Biointerfaces.

[68] Naveenkumar S., Venkateshan N., Kaviyarasu K., Christyraj J.R.S.S., Muthukumaran A. (2023). Optimum Performance of a Novel Biocompatible Scaffold Comprising Alginate-Pectin-Selenium Nanoparticles for Cardiac Tissue Engineering Using C2C12 Cells. J. Mol. Struct..

[69] El-Batal A.I., Mosallam F.M., Ghorab M.M., Hanora A., Gobara M., Baraka A., Elsayed M.A., Pal K., Fathy R.M., Elkodous M.A. (2020). Factorial Design-Optimized and Gamma Irradiation-Assisted Fabrication of Selenium Nanoparticles by Chitosan and *Pleurotus ostreatus* Fermented Fenugreek for a Vigorous In Vitro Effect against Carcinoma Cells. Int. J. Biol. Macromol..

[70] Kumar A., Sevonkaev I., Goia D.V. (2014). Synthesis of Selenium Particles with Various Morphologies. J. Colloid Interface Sci..

[71] Tang K., Yu D., Wang F., Wang Z. (2006). Controlled Growth of Hexagonal Trigonal Selenium Microtubes. Cryst. Growth Des..

[72] El-Sayed H.S., El-Sayed S.M., Youssef A.M. (2022). Designated Functional Microcapsules Loaded with Green Synthesis Selenium Nanorods and Probiotics for Enhancing Stirred Yogurt. Sci. Rep..

[73] Salman A.S., Alkhatib S.N., Ahmed F.M., Hamouda R.A. (2023). Chitosan Nanoparticles Loaded with *Capparis cartilaginea* Decne Extract: Insights into Characterization and Antigenotoxicity In Vivo. Pharmaceutics.

[74] Hassani A., Mahmood S., Enezei H.H., Hussain S.A., Hamad H.A., Aldoghachi A.F., Hagar A., Doolaanea A.A., Ibrahim W.N. (2020). Formulation, Characterization and Biological Activity Screening of Sodium Alginate-Gum Arabic Nanoparticles Loaded with Curcumin. Molecules.

[75] Pakolpakçıl A., Draczynski Z. (2021). Green Approach to Develop Bee Pollen-Loaded Alginate Based Nanofibrous Mat. Materials.

[76] Soares J.P., Santos J.E., Chierice G.O., Cavalheiro E.T.G. (2004). Thermal Behavior of Alginic Acid and Its Sodium Salt. Eclética Química.

[77] Hamouda R.A., Fauzia A.K.Q., Shahabuddin F.S., Al-Shaikh T.M., Makharita R.R. (2023). Antibacterial Activity of Ulva/Nanocellulose and Ulva/Ag/Cellulose Nanocomposites and Both Blended with Fluoride against Bacteria Causing Dental Decay. Polymers.

[78] Aparicio-Collado J.L., García-San-Martín N., Molina-Mateo J., Torregrosa Cabanilles C., Donderis Quiles V., Serrano-Aroca A., Sabater i Serra R. (2022). Electroactive Calcium-Alginate/Polycaprolactone/Reduced Graphene Oxide Nanohybrid Hydrogels for Skeletal Muscle Tissue Engineering. Colloids Surf. B Biointerfaces.

[79] Dwivedi C., Shah C.P., Singh K., Kumar M., Bajaj P.N. (2011). An organic acid-induced synthesis and characterization of selenium nanoparticles. J. Nanotechnol..

[80] Langi B., Shah C., Singh K., Chaskar A., Kumar M., Bajaj P.N. (2010). Ionic liquid-induced synthesis of selenium nanoparticles. Mater. Res. Bull..

[81] Liu Y., Wang J., Zhu P., Zhao J., Zhang C.-J., Wilkinson A.J., Cui L. (2016). Thermal Degradation Properties of Biobased Iron Alginate Film. J. Anal. Appl. Pyrolysis.

[82] Vishakha V., Abdel-Mohsen A.M., Michalicka J., White P.B., Lepcio P., Katherine L., Jančář J. (2023). Carboxymethyl Starch as a Reducing and Capping Agent in the Hydrothermal Synthesis of Selenium Nanostructures for Use with Three-Dimensional-Printed Hydrogel Carriers. R. Soc. Open Sci..

[83] Wang C., Yang C., Zhang M., Shen H. (2023). Structure of Alginate Polysaccharide Selenium-Nanoparticles and the Mechanism of Promoting Selenium Accumulation in Rice. J. Plant Nutr. Fertil..

[84] Hamouda R.A., Hussein M.H., Elhadary A.M.A., Abuelmagd M.A. (2020). Extruded Polysaccharide/Protein Matrix from Arthrospira Platensis Cultures Mediated Silver Nanoparticles Biosynthesis and Capping. Appl. Nanosci..

[85] Alagesan V., Venugopal S. (2018). Green Synthesis of Selenium Nanoparticle Using Leaves Extract of *Withania somnifera* and Its Biological Applications and Photocatalytic Activities. BioNanoScience.

[86] Serov D.A., Khabatova V.V., Vodeneev V., Li R., Gudkov S.V. (2023). A Review of the Antibacterial, Fungicidal and Antiviral Properties of Selenium Nanoparticles. Materials.

[87] Tran T.H., Le X.C., Tran T.N.M., Nguyen N.T.T., Pham B.N., Vu D. (2023). Nano Selenium–Alginate Edible Coating Extends Hydroponic Strawberry Shelf Life and Provides Selenium Fortification as a Micro-Nutrient. Food Biosci..

[88] Slavin Y.N., Bach H. (2022). Mechanisms of Antifungal Properties of Metal Nanoparticles. Nanomaterials.

[89] Tarek A., Elnasr S., Ibrahim O.M., Alhumaimess M.S., Alsohaimi I.H., El-Ossaily Y.A., Hussein M.F., Nassar A.M., Hassan H.M.A., El-Aassar M.R. (2023). Chitosan/Selenium@Olive Oil Nanocomplex Targeted Therapy for Multiple Cancers. J. Polym. Environ..

[90] El-Zayat M., Eraqi M.M., Alrefai H., El-Khateeb A.Y., Ibrahim M.A., Aljohani H.M., Aljohani M.M., Elshaer M.M. (2021). The Antimicrobial, Antioxidant, and Anticancer Activity of Greenly Synthesized Selenium and Zinc Composite Nanoparticles Using *Ephedra aphylla* Extract. Biomolecules.

[91] Khaled A.M., Othman M.S., Obeidat S.T., Aleid G.M., Aboelnaga S.M., Fehaid A., Hathout H.M.R., Bakkar A.A., Abdel A.E., El-Garawani I.M. (2024). Green-Synthesized Silver and Selenium Nanoparticles Using Berberine: A Comparative Assessment of In Vitro Anticancer Potential on Human Hepatocellular Carcinoma Cell Line (HepG2). Cells.

[92] Estevez H., Garcia-Lidon J.C., Luque-Garcia J.L., Camara C. (2014). Effects of Chitosan-Stabilized Selenium Nanoparticles on Cell Proliferation, Apoptosis and Cell Cycle Pattern in HepG2 Cells: Comparison with Other Selenospecies. Colloids Surf. B Biointerfaces.

[93] Abdelhamid A.E., Ahmed E.H., Awad H.M., Ayoub H. (2023). Synthesis and Cytotoxic Activities of Selenium Nanoparticles Incorporated Nano-Chitosan. Polym. Bull..

