# Supplementary material for: Alginate Extracted from Azotobacter chroococcum Loaded in Selenium Nanoparticles: Insight on Characterization, Antifungal and Anticancer Activities
Source: Polymers (Basel). 2024 Jul 19;16(14):2065. doi: 10.3390/polym16142065 (PMC11281124; doi:10.3390/polym16142065)
Supplement: Supplementary file 1 [file polymers-16-02065-s001.zip › polymers-3101629-supplementary.pdf]

polymers

Supplementary information

Alginate Extracted from *Azotobacter chroococcum Loaded in* Selenium Nanoparticles: Insight on Characterization, Antifungal and Anticancer Activities

Hebah A. Sindi [1], Ragaa A. Hamouda [2,3,*], Marwa S. Abdel-Hamid [3] and Nuha M. Alhazmi [1]

[1] Department of Biological Sciences, College of Science, University of Jeddah, Jeddah 21589, Saudi Arabia; sindi@uj.edu.sa (H.S.); nmalhazmi@uj.edu.sa (N.M.A.)
[2] Department of Biology, College of Sciences and Arts, Khulais, University of Jeddah, Jeddah 21959, Saudi Arabia
[3] Microbial Biotechnology Department, Genetic Engineering and Biotechnology Research Institute, University of Sadat City, Sadat City 32897, Egypt; marwa.salah@gebri.usc.edu.eg
* Correspondence: ragaahom@yahoo.com or hamouda@gebri.usc.edu.eg or rahamouda@uj.edu.sa

Nanoparticle characterization

FT-IR

The functional groups of As/Se-NPs, and Alg-Se-NCMs were inspected using a Fourier transform infrared spectrometer (FT-IR), Thermo Fisher Nicolet IS10, (Waltham, MA, USA) spectrometer; the FT-IR spectrum ranged between 400 and 4000 cm^{-1} at a resolution 4 cm^{-1}. The nanoparticles were incorporated with KBr Pellets to determine the active groups.

Transmission Electron Microscopy (TEM)

The shape and size of biosynthesized As/Se-NPs, and Alg-Se-NCMs were assessed using TEM (JEOL JSM-6510/v, Tokyo, Japan). A drop of bio-fabricated nanoparticle solution was put on the carbon-coated copper grids and kept overnight under vacuum desiccation. The carbon-coated copper grids were then loaded onto a specimen holder.

XRD Analysis

The crystallite As/Se-NPs, and Alg-Se-NCMs was evaluated using X-ray powder diffraction (XRD) (PAN Analytical X-Pert PRO, spectris plc, Almelo, The Netherlands). The nanoparticles size was inspected using Scherrer's equation. Crystal size $L = \lambda k / c\, \beta\, \theta$, where $\lambda = 0.1540$ nm, k is the constant factor of 0.91, θ = diffraction angle in radians, and β = full width at half maximum (FWHM).

Energy-Dispersive Spectroscopy

A field emission scanning electron microscope equipped with energy-dispersive spectroscopy (EDS) (JEOL JSM-6510/v, Tokyo, Japan) was used to investigate the surface morphology and element

contents of As/Se-NPs, and Alg-Se-NCMs. Nanoparticle solutions were centrifuged for 20 min at 10,000 rpm and drop-coated onto a thin glass film. The samples were then examined using scanning electron microscope compositional analysis, and the conformation of the presence of elemental selenium was carried out through energy-dispersive X-ray spectroscopy.

Zeta Potential

The zeta potential of the As/Se-NPs, and Alg-Se-NCMs solution provided details of the stabilization of the nanoparticles (Malvern zeta size nano-Zs90, Malvern, PA, USA).

Differential Scanning Calorimetry (DSC) and Thermogravimetric Analysis (TGA)

The thermal properties of As/Se-NPs, and Alg-Se-NCMs were studied by DSC and TGA. About 6mg sample was used to determine thermal analysis using TGA (TGA-50H Thermogravimetric analyzer). The sample was scanned at a flow rate of 40 mL/min over a temperature of 800 °C. The DSC technique is used to investigate the sample changes with temperature or time changes. The DSC thermogram behavior was investigated up to 400 °C.
